# Effect of Ginkgo Biloba Extract 50 on Immunity and Antioxidant Enzyme Activities in Ischemia Reperfusion Rats

**DOI:** 10.3390/molecules16119194

**Published:** 2011-11-02

**Authors:** Shaoping Lu, Xia Guo, Pinting Zhao

**Affiliations:** 1 Department of Cardiology, Tangdu Hospital, Fourth Military Medical University, Xi’an 710038, China; 2 Department of Cardiology, ChenCang Hospital, BaoJi 721300, China; Email: xiaguohp9@sina.com (X.G.); 3 Department of Radiotherapy, Tangdu Hospital, Fourth Military Medical University, Xi’an 710038, China; Email:zhaoptxatd9@sina.com (P.T.Z.)

**Keywords:** myocardial ischemia-reperfusion, antioxidant, immunity, rat, GBE50

## Abstract

The aim of the study was to investigate the effect of *Ginkgo biloba* extract 50 (GBE50), a well-known natural antioxidant, against immunity and antioxidant enzyme activities in ischemia reperfusion (IR) rats. Rats were then divided into six groups fed for 15 days with the same diet: three groups (IV, V, VI) were treated by different doses of GBE50 suspension [20, 40, or 60 mg/kg body weight by oral gavage every day at a fixed time (10.00 a.m.)] (equal to 5, 10 and 20 times, respectively, the maximum recommended human dose), and three groups (I, II, III) were untreated. At the end of the experiment, rats’ hearts were subjected to 30 min of ischemia followed by 90 min of reperfusion. Results showed that IR significantly enhanced heart rate, S-T height, myocardium (myeloperoxidase) MPO activity and blood interleukin-8 (IL-8), tumor necrosis factor Alpha (TNF-α), interleukin-1β (IL-1β) levels, blood aspartate transaminase (AST), lactate dehydrogenase (LDH), and creatinine kinase (CK) activities, reduced myocardium sodium-potassium adenosine triphosphatase (Na^+^-K^+^-ATPase), calcium-magnesium adenosine triphosphatase (Ca^2+^-Mg^2+^-ATPase) activities and antioxidant enzyme activities in IR group (III) compared to sham control group (II). Pretreatment of GBE50 markedly significantly reduced heart rate, S-T height, myocardium MPO activity and blood IL-8, TNF-α, IL-1β levels, blood AST, LDH, and CK activities, enhanced myocardium Na^+^-K^+^-ATPase, Ca^2+^-Mg^2+^-ATPase activities and antioxidant enzyme activities in IR group (II) compared to IR group (III). The results suggested that the GBE50 may reduce the oxidative stress in the reperfused myocardium, and increased immunity and antioxidant activities in IR rats.

## 1. Introduction

Myocardial ischemia-reperfusion (I/R) injury is known to occur on restoration of coronary flow after a period of myocardial ischemia usually caused by coronary heart disease. Injury of myocardium due to I/R includes cardiac contractile dysfunction, arrhythmias as well as irreversible myocyte damage, *i.e.*, myocardial necrosis [1]. In ischemia and reperfusion of the heart, oxygen derived free radicals are thought to play an important role in the genesis of tissue injury [2,3,4,5]. Many reports have demonstrated that free radical scavengers reduced free radical injury in the ischemic–reperfused heart [6,7,8,9,10,11], which supports the potential therapeutic uses of the free radical scavengers in this condition. The deleterious effects of ROS on cardiac tissue can be blocked by antioxidant enzymes such as superoxide dismutase and catalase [12,13,14,15,16]. These studies indicated that antioxidants capable of scavenging ROS, including reactive oxygen free radicals such as superoxide, hydroxyl, and peroxyl radicals, could have therapeutic advantages to treat I/R-mediated cardiac injury. Thus there is a need to understand and identify suitable antioxidant interventions to salvage the myocardium from I/R-mediated tissue damage and dysfunction.

*Ginkgo biloba* extract is widely used to improve blood flow in the treatment of vascular dementia, Alzheimer’s disease, intermittent claudication, and other vascular disorders [17]. It is reported to have antioxidant, antiplatelet, and neuroprotective activity [18,19,20]. *Ginkgo biloba* extract is used in herbal medicine and may be coadministered to patients using antiplatelet agents such as ticlopidine. Trumbeckaite *et al*. reported that *Ginkgo biloba* extract improved the rat heart mitochondrial function [21]. Chen *et al.* reported the effects of *Ginkgo biloba* extract on cation currents in rat ventricular myocytes [22]. Wu *et al*. reported that *Ginkgo biloba* extract improved coronary blood flow in healthy elderly adults [23]. The typical extract of ginkgo sold in the market is standardized to contain a minimum of 6% terpene lactones (TL), 24% flavone glycosides (FGL) and less than 5 ppm ginkgolic acids [24]. Some of the most salient health benefits attributed to this type of ginkgo extracts are for addressing medical conditions such as intermittent claudication (peripheral artery disease of legs), decreased mental vitality at old age, tinnitus, Alzheimer’s disease and depression [25]. The pharmacological activity of the ginkgo extracts is linked to two major groups of phytochemicals, the flavone glycosides (FGL) and terpene lactones [26], while ginkgolic acids are the markers (undesired compounds) for safety reasons such as their cytotoxicity [27]. *Ginkgo biloba* extract 50, self-made in China, is a new type of *Ginkgo biloba* leaf extract, and its effective components include flavonols (44%) and lactones (6%). Therefore, the present study was designed to investigate the role of *Ginkgo biloba* extract 50 in suppressing the oxidative stress and cell injury induced by ischemia-reperfusion of rat heart.

## 2. Materials and Methods

### 2.1. Materials

*Ginkgo biloba* extract 50 (GBE50, Batch No: 071008) was purchased from Shanghai XinLing Science Pharmacology Ltd. Its effective components include *Ginkgo biloba* flavonoids (>44%) (total favonol glycosides and free favonols) and lactones (>6%) (*Ginkgo biloba* lactones >3.1%, bilobalide >2.9%). GBE50 was dissolved in sodium carboxymethycellulose (dissolved in distilled water) solution for a final concentration of 10, 20, 40 mg/mL.

### 2.2. Study Protocol

Male Wistar rats, weighing 280 ± 20 g, were housed under standard environmental conditions (23 ± 1 °C, 55 ± 5% humidity and a 12 h light/dark cycle) and maintained with free access to water and a standard diet *ad libitum*. The general guidelines for the care and use of laboratory animals recommended by China were followed. Rats were then divided into six groups fed for 15 days with the same diet: three groups (IV, V, VI) were treated by different doses of GBE50 suspension (20, 40, or 60 mg/kg body weight) by oral gavage every day at a fixed time (10:00 a.m.), and three groups (I, II, III) were untreated. Usage dose of GBE50 in each group was 5 mL. Diets and tap water were freely available. On the 16th day, 2 h after the above treatments, the rats in groups II-VI were subjected to the following evaluation tests.

### 2.3. In Vivo Studies of Myocardial Ischemia–Reperfusion Surgical Preparation

The ischemia–reperfusion injury was produced in rat heart based on Buerke’s description with modifications [28,29]. In brief, rats were anesthetized with pentobarbital sodium (70 mg/kg body weight) by an i.p. injection of a mixture of 20% Dorminal (1 mL contains 200 mg pentobarbital sodium, Alfasan) and sterile saline at a ratio of 1:3 (v/v). Additional doses (40 mg/kg/h) were administered continuously via the jugular vein by a micro-injection pump at intervals of approximately 2 h or at a rate as required to maintain anesthesia. After ensuring sufficient depth of anesthesia (showing as loss of palperbral reflex) the rats were placed on a warm board to control the body temperature at 37 °C for surgery. The neck was opened with a ventral midline incision. The trachea was exposed and cannulated with a PE-90 catheter to establish artificial respiration provided by a SAR-830/P ventilator (IITC, USA) with oxygen at a breath ratio of 1:1 and at a frequency of 70–80 breaths/min with tidal volume of 0.8–1.2 mL. The right carotid artery was isolated and a Millar catheter (Millar Instruments, Inc., Houston, TX, USA) was inserted into the right carotid artery. The electrocardiogram (ECG) in lead II was recorded through the needle electrodes attached to the limbs. The heart rate and ST-segment elevation were calculated off-line. The chest was opened at the left fourth intercostal space. The pericardium was incised and the left atrium appendage was elevated to expose the left anterior descending (LAD) coronary artery. A 6-0 silk suture was passed around the LAD coronary artery, and the ends of suture were threaded through a small vinyl tube to form a snare. The thoracic cavity was covered with saline-soaked gauze to prevent the heart from drying. At 1 h after drug administration, ischemia was established by tightening the suture from both ends with fixed weight. The animals then underwent 30 min of ischemia, confirmed visually *in situ* by the appearance of regional epicardial cyanosis and ST-segment elevation. Reperfusion was introduced by releasing the snare gently for a period of 90 min. The sham control animals were subjected to the entire surgical procedure described above, except the introduction of LAD ligation and release. At the end of reperfusion, blood samples were drawn from the abdominal aortic artery and sera were prepared by centrifuging at 1,000 × g for 10 min at room temperature. The sera were stored at −80 °C until the measurement of biochemical indexes. After collection of serum samples, 2 mL of ice-cold 10% potassium chloride was injected via inferior vena cava to stop the heart in diastole and the heart was excised and weighed immediately. The heart was frozen in liquid nitrogen before being stored at −80 °C.

### 2.4. Biochemical Analysis

Measurement of myeloperoxidase activity (MPO): Tissue samples (heart) were homogenized in ice-cold 50 mM potassium phosphate buffer pH-6 containing 0.5% hexadecyltrimethylammonium bromide (HTAB, Sigma). The homogenate was freeze thawed three times then centrifuged at 11,000 × g for 20 min at 4 °C. The supernatant (34 μL) was mixed with the same phosphate buffer (986 μL) containing 0.167 mg mL^−1^ *ortho*-dianisidine dihydrochloride (Sigma) and 0.0005% hydrogen peroxide. The change in absorbance at 460 nm was recorded by spectrophotometer. One unit of MPO activity was defined as that consuming 1 nmol of peroxide per minute at 22 °C. The results were expressed as unit (g protein)^−1^ [30].

The serum was separated after blood collection for the estimation of creatine kinase activity using Span Diagnostic kits.

IL-8, IL-1β and TNF-α levels were measured using commercial enzyme immunoassay kits (Endogen, Woburn, MA, USA).

Aspartate aminotransferase (AST) and lactate dehydrogenase (LDH) were measured in serum using an Automated Chemical Analyzer (Bayer, Leverkusen, Germany).

Na^+^-K^+^-ATPase and Ca^2+^-Mg^2+^-ATPase activities were assayed by spectrophotometrically measuring the amount of inorganic phosphate liberated following incubation of the tissue extract with disodium ATP (Sigma, England) as in previous studies [31]. Specific activity of the enzyme was expressed as nmol Pi released per min per mg of protein (μmol Pi/hour/mg of protein).

Lipid peroxidation was estimated by measuring thiobarbituric acid-reactive substances (TBARS) and expressed in terms of malondialdehyde (MDA) content, according to the method of Draper and Hadley [32]. The MDA values were calculated using 1,1,3,3-tetraethoxypropane as standard and expressed as nmol of MDA/g pancreas.

GSH was measured through a reaction using dithiobisnitrobenzoic acid (DTNB), a symmetric aryl disulfide. DTNB reacts with the free thiol to give a mixed disulfide plus 2-nitro-5-thiobenzoic acid (TNB), which is quantified by its absorbance at 412 nm [33].

Superoxide dismutase (SOD) activity was estimated according to the literatures [34,35]. The developed blue colour in the reaction was measured at 560 nm. Units of SOD activity were expressed as the amount of enzyme required to inhibit the reduction of NBT by 50% and the activity was expressed as U/mg protein.

CAT activity was measured using the method of Regoli and Principato [36]. Twenty microlitres of the supernatant was added to a cuvette containing 780 μL of a 50 M potassium phosphate buffer (pH 7.4), and then the reaction was initiated by adding 200 μL of 500 mM H_2_O_2_ to make a final volume of 1.0 mL at 25 °C. The decomposition rate of H_2_O_2_ was measured at 240 nm for 1 min on a spectrophotometer. A molar extinction coefficient of 0.0041 mM^−1^cm^−1^ was used to determine the catalase activity. The activity was defined as the μ mole H_2_O_2_ decrease/mg protein/min.

The activity of GSH-Px was determined by quantifying the catalyzed reaction rate of H_2_O_2_ and GSH. One unit (U) of GSH-px was defined as the amount that reduced the level of GSH by 1 μmol/(L mg) protein.

Glutathione S-transferase (GST; EC 2.5.1.18) catalyzes the conjugation reaction with glutathione in the first step of mercapturic acid synthesis. The activity of GST was measured according to the method of Habig *et al.* [37]. *p*-Nitrobenzyl chloride was used as substrate. The absorbance was measured spectrophotometrically at 310 nm using a UV-double beam spectrophotometer.

### 2.5. Histopathologic Detection

After 12 h in 4% paraformaldehyde, heart samples were dehydrated and embedded in paraffin, cut into 4 μm slices, heated overnight in a 60 °C incubator, and then dewaxed and stained with H&E and Masson dye. One slice was chosen from each rat and was analyzed under a microscope. Photomicrographs were taken using Olympus SZX7 zoom stereo microscope or Olympus BX51 microscope plus Olympus DP71 CCD camera (Olympus Corporation, Tokyo, Japan).

### 2.6. Statistical Analyses

All results were expressed as mean ± SD and were analyzed by SPSS for Windows, version 11.5 (SPSS Inc, Chicago, IL, USA). Data were subjected to one-way analysis of variance (ANOVA) followed by multiple comparison with the Student-Newman-Keuls test. For all comparisons, a value of P less than 0.05 was considered statistically significant.

## 3. Results

Sham-operated (group II) rat hearts revealed normal myofibril structural architecture without any fraying or infarction. In IR group (III), histopathological section showed severe necrotic patches of myocardial tissue along with interstitial edem. In addition focal confluent necrosis of muscle fibers with inflammatory cell infiltration, vacuolar degeneration and myophagocytosis along with extravasation of red blood cells was also observed. This indicated that myocardium IR rats model had been successfully established.

Acute myocardial I/R injury causes ST-segment elevation, arrhythmia, and hemodynamic changes in rats. As shown in Table 1, there wasn’t marked difference (*p* > 0.05) in heart rate and ST height between group I and group II. In our experiments, occlusion of the LAD coronary artery resulted in a significant heart rate and ST-segment elevation at 30 min after ischemia in group III compared to group II (*p* < 0.05, *p* < 0.01, Table 1). Pretreatment with GBE50 suspension at 50, 100, or 200 mg/kg body weight significantly inhibited the heart rate and ST-segment elevation in group IV, V, VI rats compared to group III (*p* < 0.05, *p* < 0.01, Table 1). In drug (GBE50 suspension) treated group, there were only focal necrosis with mild interstitial edema and lesser inflammatory cell infiltration as compared to IR group. In addition, there wasn’t significant difference in body weight between all groups of rats (*p* > 0.05, Table 1).

**Table 1:** Effect of GBE50 on heart rate and S-T height in rats.

Group	Body weight (g)	HR beat/min	S-T height (mv)
I	313.1 ± 29.7	422 ± 12	0.05 ± 0.03
II	315.5 ± 35.2	426 ± 43	0.05 ± 0.02
III	316.9 ± 33.8	441 ± 28 ^a^	0.6 ± 0.05 ^b^
IV	310.4 ± 36.2	432 ± 54	0.32 ± 0.03 ^d^
V	312.9 ± 30.8	429 ± 33 ^c^	0.24 ± 0.02 ^d^
VI	316.1 ± 31.7	429 ± 38 ^c^	0.11 ± 0.02 ^d^

^a^ *p* < 0.05; ^b^ *p* < 0.01, compared with group II; ^c^ *p* < 0.05; ^d^ *p* < 0.01, compared with group III.

As shown in Table 2, there was no marked difference in myocardium MPO activity and blood IL-8, IL-1β levels between group I and group II. Blood TNF-ɑ level in group II is markedly (*p* < 0.05) higher than that in group I. The myocardium MPO activity and blood IL-8, TNF-α, IL-1β levels of the group III rats remained significantly high throughout the experiment periods compared to those of group II rats. The pre-administration of GBE50 suspension at 50, 100, or 200 mg/kg body weight significantly to rats reduced myocardium MPO activity and blood IL-8, TNF-α, IL-1β levels (*p* < 0.01).

**Table 2:** Effect of GBE50 on myocardium MPO activity and blood IL-8, TNF-α, IL-1β levels in rats.

Group	MPO (U/g)	IL-8 (ng/mL)	TNF-ɑ (ng/mL)	IL-1β (ng/mL)
I	0.3 ± 0.02	0.71 ± 0.05	1.02 ± 0.17	0.42 ± 0.04
II	0.31 ± 0.04	0.75 ± 0.06	1.21 ± 0.11 ^e^	0.41 ± 0.03
III	1.14 ± 0.21 ^b^	1.93 ± 0.23 ^b^	2.18 ± 0.18 ^b^	0.96 ± 0.08 ^b^
IV	0.89 ± 0.07 ^d^	1.52 ± 0.16 ^d^	1.78 ± 0.14 ^d^	0.71 ± 0.06 ^d^
V	0.57 ± 0.06 ^d^	1.12 ± 0.13 ^d^	1.42 ± 0.15 ^d^	0.53 ± 0.04 ^d^
VI	0.42 ± 0.03 ^d^	0.88 ± 0.06 ^d^	1.26 ± 0.14 ^d^	0.45 ± 0.05 ^d^

^e^ *p* < 0.05, compared with group I; ^b^ *p* < 0.01, compared with group II; ^d^ *p* < 0.01, compared with group III.

When blood AST, LDH, and CK activities in rats pre-treated with and without GBE50 suspension were determined after IR treatment, the results shown in Table 3 were obtained. Although blood AST, LDH, and CK activities in group II rats was higher than those in group I rats, difference wasn’t significant (*p* > 0.05). Blood AST, LDH, and CK activities in group III rats were significantly (*p* < 0.01) higher than those in group II rats. Pre-administration of GBE50 suspension (50, 100, or 200 mg/kg body weight) significantly (*p* < 0.01) increased the blood AST, LDH, and CK activities in IR rats (group IV-VI) in comparison to group III rats.

**Table 3:** Effect of GBE50 on blood AST, LDH, and CK activities in rats.

Group	AST (U/L)	LDH (U/L)	CK (U/L)
I	491.4 ± 32.7	322.3 ± 20.6	483.1 ± 35.2
II	507.4 ± 39.8	325.1 ± 28.5	504.7 ± 44.1
III	1438.1 ± 132.6 ^b^	872.9 ± 57.7 ^b^	938.6 ± 73.9 ^b^
IV	1118.4 ± 121.7 ^d^	705.4 ± 45.2 ^d^	794.2 ± 62.1 ^d^
V	894.6 ± 56.6 ^d^	573.1 ± 43.9 ^d^	605.3 ± 47.8 ^d^
VI	603.2 ± 43.7 ^d^	397.4 ± 30.54 ^d^	512.6 ± 40.5 ^d^

^b^ *p* < 0.01, compared with group II; ^d^ *p* < 0.01, compared with group III.

The heart tissue was used to determine the protective effect of GBE50 (Table 4). Myocardium Na^+^-K^+^-ATPase and Ca^2+^-Mg^2+^-ATPase activities in group II were insignificantly (*p* > 0.05) decreased compared to those of normal control group (I). Compared with those of the sham group (II), myocardium Na^+^-K^+^-ATPase and Ca^2+^-Mg^2+^-ATPase activities in group III were significantly decreased (*p* < 0.01). Different doses (50, 100, or 200 mg/kg body weight) of GBE50 significantly (*p* < 0.05, *p* < 0.01) increased the myocardium Na^+^-K^+^-ATPase and Ca^2+^-Mg^2+^-ATPase activities in a dose-dependent manner compared with those of group III (Table 4).

**Table 4:** Effect of GBE50 on myocardium Na^+^-K^+^-ATPase and Ca^2+^-Mg^2+^-ATPase activities in rats.

Group	Na^+^-K^+^-ATPase (μmol Pi/mg prot/hour)	Ca^2+^-Mg^2+^-ATPase (μmol Pi/mg prot/hour)
I	9.98 ± 0.64	7.53 ± 0.62
II	9.27 ± 0.84	7.49 ± 0.57
III	4.01 ± 0.37 ^b^	3.51 ± 0.31 ^b^
IV	6.32 ± 0.41 ^d^	5.08 ± 0.39 ^c^
V	8.05 ± 0.66 ^d^	6.73 ± 0.44 ^d^
VI	9.86 ± 0.73 ^d^	7.55 ± 0.53 ^d^

^b^ *p* < 0.01, compared with group II; ^c^ *p* < 0.05, ^d^ *p* < 0.01, compared with group III.

Compared with that of the normal control group (I), the levels of myocardium MDA and GSH in group II rats were slightly (*p* > 0.05) increased or decreased (Table 5). The level of myocardium MDA in group III rats was significantly (*p* < 0.01) increased, whereas level of myocardium GSH was significantly (*p* < 0.01) decreased compared to those of group II. As shown in Table 5, pre-treatment of GBE50 suspension (50, 100, or 200 mg/kg body weight) dose-dependently significantly (*p* < 0.01) decreased the myocardium MDA content and increased myocardium GSH levels in groups IV-VI compared to group III.

**Table 5:** Effect of GBE50 on myocardium MDA and GSH levels in rats.

Group	MDA (nmol/mg protein)	GSH (mg/g protein)
I	4.78 ± 0.26	139.8 ± 11.8
II	5.06 ± 0.41	121.7 ± 10.4
III	9.38 ± 0.68 ^b^	65.3 ± 4.2 ^b^
IV	7.12 ± 0.62 ^d^	85.9 ± 5.8 ^d^
V	6.37 ± 0.57 ^d^	108.5 ± 7.4 ^d^
VI	5.22 ± 0.38 ^d^	133.4 ± 9.7 ^d^

^b^ *p* < 0.01, compared with group II; ^d^ *p* < 0.01, compared with group III.

Compared with that of the normal control group (I), the activities of myocardium SOD, CAT, and GSH-Px in group II rats were slightly (*p* > 0.05) decreased, whereas GST activity in group II rats was significantly (*p* < 0.05) reduced (Table 6). The activities of myocardium SOD, CAT, GSH-Px and GST in group III rats were significantly (*p* < 0.01) decreased compared to those of group II. As shown in Table 6, pre-treatment of GBE50 (50, 100, or 200 mg/kg body weight) dose-dependently significantly (*p* < 0.05, *p* < 0.01) increased the activities of myocardium SOD, CAT, GSH-Px and GST in groups IV-VI compared to group III (Table 6).

**Table 6:** Effect of GBE50 on myocardium SOD, CAT, GSH-Px and GST activities in rats.

Group	SOD	CAT	GSH-Px	GST
	(U/mg protein)	(U/mg protein)	(U/mg protein)	(U/mg protein)
I	328.6 ± 21.7	29.87 ± 1.07	36.13 ± 1.64	14.76 ± 1.09
II	311.8 ± 19.7	26.73 ± 1.36	33.12 ± 1.83	11.87 ± 0.97 ^e^
III	157.3 ± 11.1 ^b^	12.68 ± 1.22 ^b^	20.43 ± 1.24 ^b^	5.73 ± 0.42 ^b^
IV	198.4 ± 13.5 ^c^	18.43 ± 1.05 ^c^	27.49 ± 1.93 ^d^	7.84 ± 0.63 ^d^
V	259.1 ± 19.8 ^d^	25.16 ± 1.53 ^d^	33.12 ± 1.68 ^d^	10.52 ± 0.83 ^d^
VI	305.7 ± 20.3 ^d^	30.76 ± 1.7 ^d^	36.05 ± 1.82 ^d^	12.93 ± 0.74 ^d^

^e^ *p* < 0.05, compared with group I; ^b^ *p* < 0.01, compared with group II; ^c^ *p* < 0.05; ^d^ *p* < 0.01, compared with group III.

## 4. Discussion

There were some previous studies related to GBE50 heart protection activities. Want *et al*. reported that GBE50 has inhibitory effects on DADs and TA induced by ouabain and high Ca^2+^ in guinea pig papillary muscles [38]. Bao *et al*. reported that GBE50 could relieve arrhythmia following I/R and alleviate I/R injury possibly by inhibiting free radicals [39]. Zhang *et al*. reported that GBE50 could promote expression of the antiapoptotic gene bcl-2 and bcl-xL in rabb itmyocardium [40]. In the present study, we investigated the effect of GBE50 on I/R-induced cardiac injury. Administration of GBE50 at the onset of reperfusion improved the physiological functions of the heart as a result of the increased HR and decreased S-T height. Acute injury in the form of ischemia leading to production of inflammatory mediators precipitates into myocardial functional suppression [41,42]. MPO, a polymorpho nuclear neutrophil derived heme protein, is a biomarker index for neutrophil infiltration and inflammatory reactivity. Role of MPO activity in myocardial reperfusion injury is well established [43,44]. The MPO activity in the present study showed steep increment in I/R animals. This indicated that white blood cell count was sharply increasing in ischemia myocardium tissue.

Conclusive evidence demonstrates the involvement of proinflammatory cytokines such as tumor necrosis factor (TNF)-α, interleukin-1β and interleukin-8 in the postischemic response. This is corroborated by the finding, that both defective IL-1β signaling [45] as well as TNF-α signaling [46] resulted in decreased chemokine upregulation and attenuated neutrophil infiltration and that in a selection of patients with episodes of ischemia/reperfusion (major blunt trauma, ruptured aortic aneurysm) increased levels of TNF-α, IL-1β and IL-8 are associated with increased mortality and increased risk for acute respiratory distress syndrome and multi-organ failure [47,48]. In the present study, blood TNF-α, IL-1β and IL-8 levels were found to significantly increased compared to the sham group. However, treatment with GBE50 significantly decreased myocardium MPO activity and blood IL-8, TNF-α, IL-1β levels in IR rats. This indicated that GBE50 could reduce inflammation in ischemia ischemia myocardium tissue.

IR doesn’t only destroy myocardial cell structure, but also induces outleakage of intracellular dehydrogenase and marker enzymes. Our results show significant elevation in the levels of diagnostic marker enzymes (AST, LDH and CK) in serum of IR rats, which is in line with an earlier report [49], and is an indication of the severity of IR-induced necrotic damage of the myocardial membrane. In the present study, the prior administration of GBE50 was found to significantly lower the IR-induced elevation in the levels of diagnostic marker enzymes in groups IV-VI, which indicates the decreased outleakage of intracellular dehydrogenase and marker enzymes.

ATPase enzymes in the membrane are responsible for ionic pump regulation. Myocardial cell ATPases like Na^+^-K^+^-ATPase, Mg^2+^-Ca^2+^-ATPase are critical for normal myocardial cell functions such as exchanges of intra- and extra-cellular electrolyte homeostasis and membrane integrity. Our results indicate that the activity of myocardial Na^+^-K^+^-ATPase decreased significantly during ischemia reperfusion. A similar pattern of changes was shown by other ATPase like Ca^2+^-Mg^2+^-ATPase. Pre-treatment of GBE50 could enhance myocardial Na^+^-K^+^-ATPase and Ca^2+^-Mg^2+^-ATPase activities. This further confirmed cardioprotective effect of GBE50.

Although IR-induced myocardial injury occurs due to the complex interaction of numerous factors, increased production of ROS plays a major role in IR-induced cardiac injury. Hence, it is not surprising that overexpression of myocardial antioxidants or the introduction of a mitochondrial-targeted antioxidant minimizes IR-induced myocardial injury [50,51]. Further, dietary supplementation with antioxidants has been shown to lower IR-induced myocardial oxidative injury and the magnitude of infarction [52,53].

The present results showed some indications of protection against oxidative stress. There was significant difference for TBARS and total glutathione between the IR group and the sham control group. Pre-treatment of GBE50 could reduce myocardial TBARS and enhance total glutathione levels, indicating a tendency toward protection. Moreover, with a larger dose of GBE50 the difference between the IR group and GBE50-treated group in markers of oxidative stress in the current protocol may have reached significance, in accordance with these previous studies that showed protection of heart against oxidative stress with higher doses [54,55].

Since the disruption of endogenous antioxidant network due to I/R was also shown in the previous studies, the myocardium may be more susceptible to the damage [56,57,58]. Antioxidant enzymes play an important role in the ROS cascade reaction: superoxide dismutase (SOD) transforms superoxide radical (O_2_^−^) into hydrogen peroxide (H_2_O_2_), catalase (CAT) and glutathione peroxidase (GPx) remove hydrogen peroxide and limit the hydroxyl radical (OH^−^) formation, and glutathione-S-transferases (GST) removes toxic products of ROS damage [59]. In the present study, SOD, CAT, GSH-Px and GST activities as endogenous antioxidant enzymes were significantly decreased in the hearts of IR group than those of the sham control group after I/R. Pre-treatment of GBE50 dose-dependently significantly increased myocardium SOD, CAT, GSH-Px and GST activities in GBE50-treated rats compared to the sham control group. The I/R-induced injury depresses cardiac function because of the loss of active cardiomyocytes caused by increased free radical production. GBE50 might scavenge the free radicals and enhance myocardium antioxidant enzyme activities.

## 5. Conclusions

Collectively, these studies demonstrate the importance of myocardial antioxidant capacity in providing protection against IR-induced injury.
